# Supplementary material for: The interactome of intact mitochondria by cross-linking mass spectrometry provides evidence for coexisting respiratory supercomplexes
Source: Mol Cell Proteomics. 2017 Dec 8;17(2):216–32. doi: 10.1074/mcp.RA117.000470 (PMC5795388; doi:10.1074/mcp.RA117.000470)
Supplement: Supplemental Data [file supp_RA117.000470_133922_0_supp_23978_fzffwf.zip › spectra_annotation/mito_DR_spectra_annotation/455-1-8-1-12-1.pdf]

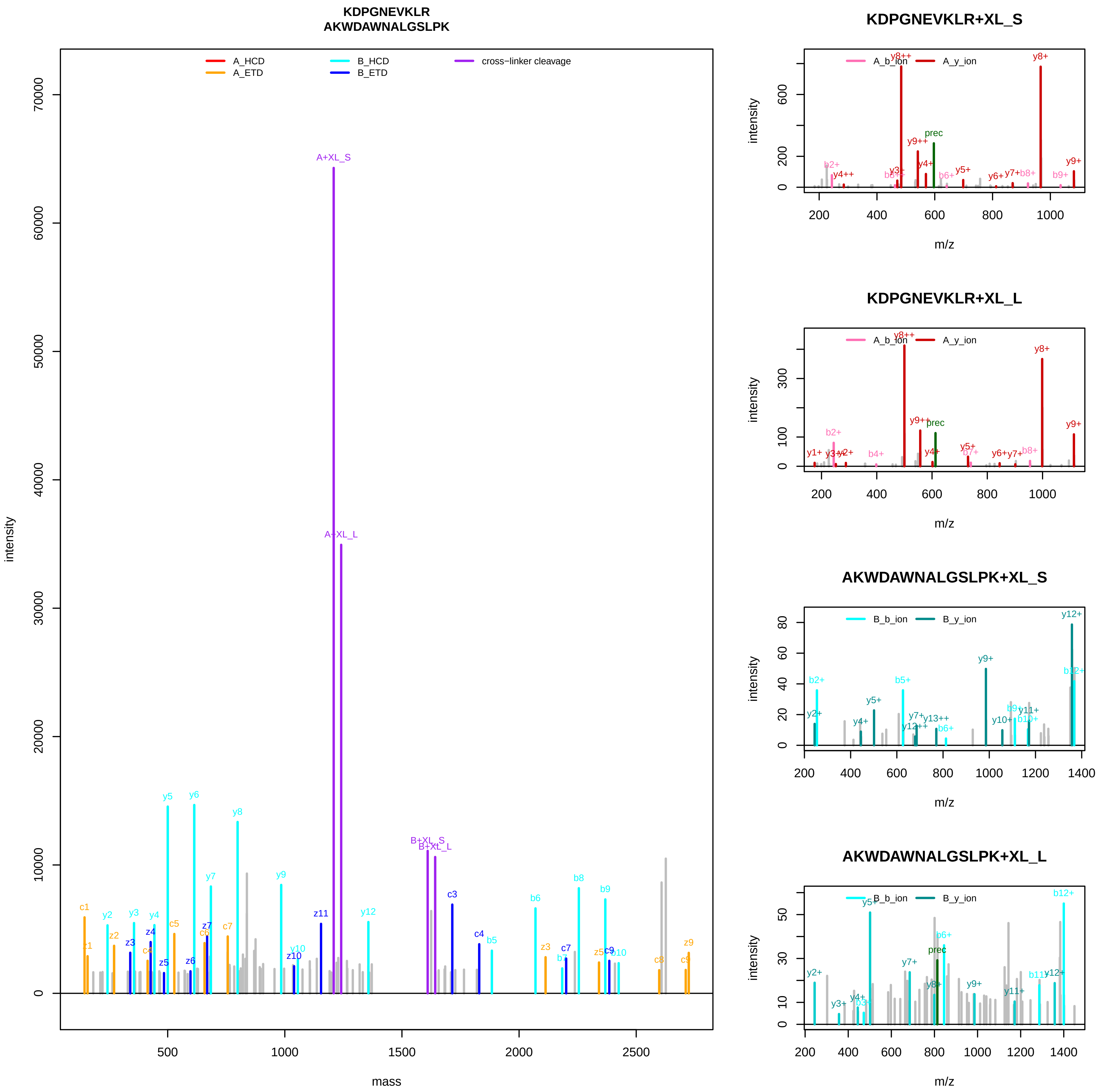
KDPGNEVKLR
AKWDAWNALGSLPK
A_HCD
A_ETD
B_HCD
B_ETD
cross−linker cleavage
intensity
mass
0
10000
20000
30000
40000
50000
60000
70000
500
1000
1500
2000
2500
A+XL_S
A+XL_L
B+XL_S
B+XL_L
c1 z1 y2 z2 y3 z3 y4 z4 c4 y5 z5 c5 y6 z6 c6 z7 y7 c7 y8 y9 y10 z10 z11 y12 c3 c4 b5 b6 z3 b7 c7 b8 z5 b9 c9 b10 c8 c9 z9
KDPGNEVKLR+XL_S
A_b_ion
A_y_ion
y8++ y8+ y9++ prec b2+ y4++ y3+ b5+ y4+ y5+ b6+ y6+ y7+ b8+ b9+ y9+
KDPGNEVKLR+XL_L
A_b_ion
A_y_ion
y8++ y8+ y9++ prec b2+ y1+ y3+ y2+ b4+ y4+ y5+ b7+ y6+ y7+ b8+ y9+
AKWDAWNALGSLPK+XL_S
B_b_ion
B_y_ion
y12+ b12+ y9+ b2+ b5+ y5+ y2+ y4+ y7+ y13++ y12++ b6+ b9+ y11+ y10+ b10+
AKWDAWNALGSLPK+XL_L
B_b_ion
B_y_ion
b12+ y5+ b6+ prec y7+ y2+ y8+ y9+ y11+ b11+ y12+ y3+ y4+ b3+
intensity
m/z
